# Comparative analysis of pulmonary function in children born preterm and full-term at 6–9 years of age

**DOI:** 10.1590/1984-0462/2023/41/2021294

**Published:** 2022-09-09

**Authors:** Ana Damaris Gonzaga, Josy Davidson, Ana Lucia Goulart, Marina Carvalho de Moraes Barros, Sonia Mayumi Chiba, Amélia Miyashiro Nunes dos Santos

**Affiliations:** a Universidade Federal de São Paulo, São Paulo, SP, Brazil.

**Keywords:** Child, Premature birth, Lung function tests, Spirometry, Criança, Nascimento prematuro, Testes de função pulmonar, Espirometria

## Abstract

**Objective:**

To compare pulmonary function parameters and the prevalence of altered pulmonary function in children born preterm and full-term, using the Global Lung Initiative reference values.

**Methods:**

This is a cross-sectional study with 6–9-year-old children submitted to measurement of airway resistance (Rint) and spirometry according to the *American Thoracic Society and European Respiratory Society Technical Statement*. The inclusion criteria were, among the preterm group: gestational age <37 weeks and birth weight <2000g; among the full-term group: schoolchildren born full-term with birth weight >2500g, recruited at two public schools in São Paulo, Brazil, matched by sex and age with the preterm group. As exclusion criteria, congenital malformations, cognitive deficit, and respiratory problems in the past 15 days were considered.

**Results:**

A total of 112 children were included in each group. Preterm children had gestational age of 30.8±2.8 weeks and birth weight of 1349±334g. Among them, 46.6% were boys, 46.4% presented respiratory distress syndrome, 19.6% bronchopulmonary dysplasia, and 65.2% were submitted to mechanical ventilation in the neonatal unit. At study entry, both groups were similar in age and anthropometric parameters. Parameters of pulmonary function (Z scores) in preterm and full-term groups were: Rint (0.13±2.24 vs. -1.02±1.29; p<0.001); forced vital capacity (FVC) (-0.39±1.27 vs. -0.15±1.03; p=0.106), forced expiratory volume in one second (FEV_1_)/FVC (-0.23±1.22 vs. 0.14±1.11; p=0.003), FEV_1_ (-0.48±1.29 vs. -0.04±1.08; p=0.071), and forced expiratory flow between 25% and 75% of vital capacity (FEF_25-75_) (1.16±1.37 vs. 2.08±1.26; p=0.005), respectively. The prevalence values of altered airway resistance (16.1 vs. 1.8%; p<0.001) and spirometry (26.8 vs. 13.4%, p=0.012) were higher in preterm infants than in full-term ones.

**Conclusions:**

Preterm children had higher prevalence of altered pulmonary function, higher Z scores of airway resistance, and lower Z scores of FEV_1_/FVC and FEF_25-75_ compared with those born full-term.

## RESUMO

**Objetivo:**

Comparar parâmetros de função pulmonar e a prevalência de função pulmonar alterada em crianças nascidas pré-termo e a termo, utilizando a referência Global Lung Function Initiative.

**Métodos:**

Estudo transversal com crianças de 6–9 anos submetidas à medida de resistência de vias aéreas (Rint) segundo o American Thoracic Society and the European Respiratory Society Technical Statement. Como critérios de inclusão, entre o grupo pré-termo, estavam os nascidos com idade gestacional <37 semanas e peso <2000g; e entre o grupo termo, escolares de duas escolas públicas do município de São Paulo, nascidos a termo com peso >2500g, pareados por sexo e idade com o grupo pré-termo. Excluíram-se malformações congênitas, déficit cognitivo e problemas respiratórios havia menos de 15 dias.

**Resultados:**

Incluíram-se 112 crianças em cada grupo. Os prematuros (46,4% masculinos) apresentaram idade gestacional de 30,8±2,8 semanas e peso de 1349±334g. Entre eles, 46,4% tiveram síndrome de desconforto respiratório, 19,6% displasia broncopulmonar, e 65,2% receberam ventilação mecânica na unidade neonatal. À inclusão no estudo, os dois grupos apresentaram idade e dados antropométricos semelhantes. Os valores (escores Z) em nascidos pré-termo e a termo foram, respectivamente: Rint (0,13±2,24 *vs*. -1,02±1,29; p<0,001), capacidade vital forçada (CVF) (-0,39±1,27 *vs*. -0,15±1,03; p=0,106), volume expiratório forçado no primeiro segundo (VEF_1_)/CVF (-0,23±1,22 *vs*. 0,14±1,11; p=0,003), VEF_1_ (-0,48±1,29 *vs*. -0,04±1,08; p=0,071) e fluxo expiratório forçado em 25–75% da capacidade vital (FEF_25-75_) (1,16±1,37 *vs*. 2,08±1,26; p=0,005). A prevalência de alterações na resistência de vias aéreas (16,1 *vs*. 1,8%, p<0,001) e na espirometria (26,8 *vs*. 13,4%, p=0,012) foi maior nos prematuros.

**Conclusões:**

As crianças nascidas pré-termo apresentaram maior prevalência de alteração pulmonar, maiores escores Z de resistência de vias aéreas e menores escores Z de VEF_1_/CVF e FEF_25-75_ quando comparadas às nascidas a termo.

## INTRODUCTION

Studies show that children, adolescents, and even adults born preterm may present alterations in pulmonary function, with decreased forced vital capacity (FVC), forced expiratory volume in one second (FEV_1_), and forced expiratory flow between 25% and 75% of vital capacity (FEF_25-75_).^1–5^

It is unclear whether differences found in pulmonary function tests in individuals born preterm are inherent in the premature interruption of pulmonary development or whether they are consequences of maternal and neonatal factors associated with prematurity, especially the occurrence of bronchopulmonary dysplasia.^6,7^ In this sense, Yang et al.^7^ found significantly lower values of Z scores for FVC, FEV_1_/FVC, FEV_1_, and FEF_25-75_ in adults aged 26 to 30 years born preterm with or without bronchopulmonary dysplasia.

In addition, the use of a new reference curve, the Global Lung Function Initiative (GLI), considered as the universal gold standard, has contributed to differences in the results of pulmonary function tests in children and adults born preterm.^8^

Thus, Thunqvist et al.^5^ described, using GLI standards for 6.5-year-old children born with gestational age <27 weeks, the prevalence of altered pulmonary function with Z scores <-1.64 of 14 and 23% for FVC and FEV_1_, respectively. Comparatively, Gonçalves et al.,^3^ using the Polgar and Promadhat’s curve,^9^ observed a much higher prevalence of altered pulmonary function in children and adolescents born with a mean of 32 weeks of gestational age, reflecting the influence of the curve used in the assessment of pulmonary function.

In this context, the present study aimed to compare the prevalence of altered pulmonary function and to analyze pulmonary function parameters in schoolchildren born preterm and full-term, using the GLI curve as reference.^8^

## METHOD

This is a cross-sectional study with two groups of schoolchildren aged 6 to 9 years, one group of preterm infants and a control group of full-term infants, submitted to the pulmonary function test, carried out from December 6, 2016 to March 8, 2019.

The preterm group included children with gestational age <37 weeks at birth and weight <2000g, born in the neonatal units of Universidade Federal de São Paulo (Unifesp), in São Paulo, Brazil, and selected from the follow-up list of the Premature Outpatient Clinic of the aforementioned institution. After discharge from the neonatal unit, preterm infants are routinely referred to the Premature Outpatient Clinic, where they are followed up until the end of adolescence. During the follow-up period, they are regularly assisted by a team of pediatricians, neuropediatricians, physiotherapists, speech therapists, ophthalmologists, psychologists, psychopedagogues, dentists, nutritionists, and social workers.

The control group included children of the same age, born full-term, weighing >2500g, matched by age and sex with the preterm group and recruited from two public schools in the city of São Paulo, according to the researcher’s convenience.

Initially, the list of children born preterm who met the inclusion criteria was compiled. Once having this list, the authors selected the first schoolchildren on the attendance list that could be matched by age and sex with each child in the preterm group.

In both groups, children with congenital malformations, neuromuscular disorders, and cognitive deficits that limited the understanding of the guidelines for performing pulmonary function were excluded, in addition to those with respiratory problems, such as respiratory infections, wheezing, or seeking emergency room for symptoms less than 15 days before the test.

The clinical history regarding the birth and follow-up of preterm infants was collected from the medical record and supplemented with interviews with the children’s parents. For the full-term group, a questionnaire was sent to be completed by the parents. The Fenton and Kim’s curve was used to classify the neonatal population^10^. The *International Study of Asthma and Allergies in Childhood* (ISAAC) questionnaire was used to diagnose childhood asthma.^11^

Demographic and clinical data collection and pulmonary function tests were performed on the same day for each child included in the study.

Parents/guardians of the children were instructed to discontinue medications known to influence bronchial responsiveness 24–48 hours before the test, as recommended worldwide.^12,13^

All pulmonary function measurements were taken by the same researcher, using the interrupter technique (Rint) to measure airway resistance and spirometry measures. The ROCC Pony FX^®^ portable equipment (Cosmed, Rome, Italy) was used following the international recommendations of the *American Thoracic Society and the European Respiratory Society Technical Statement* (ATS/ERS).^13^ The tests took place with children comfortably seated, using a 3cm-diameter disposable mouthpiece and nose clip.

In the interrupter technique,^14^ the flow was measured during breathing at tidal volume, with the shutter automatically closed after 10ms of the peak expiratory flow, remaining closed until 100ms. Pressure was measured using two points in the linear back-extrapolation method. Seven airflow interruptions were performed at the unforced peak expiratory flow, with random and automatic frequencies. The measurement was considered successful if at least five appropriate measures were obtained as well as the coefficient of variation of the measures ≤20%.

Measures in which air escaped through the mouthpiece, excessive neck extension or flexion, irregular breathing pattern, movement during closure of the shutter, coughing, swallowing, or sneezing were excluded.

For spirometry measures^15^, the flow/volume and volume/time curves were analyzed and excluded if they were visibly inadequate according to the reference standard. The allowed extrapolated volume was <15mL or <5% of the FVC (whichever was greater). The test ended when the volume/time curve reached the plateau and the child was unable to continue exhaling, that is, there was no change in volume per second. Reproducibility was assessed using two acceptable curves in which the second highest FVC and FEV_1_ values were <150mL.^15^

After spirometry measurements, the children were submitted to inhalation with aerosol with bronchodilator (salbutamol, 400mcg) using a spacer device (Flumax^®^), and four puffs were sprayed to reach the recommended dose of medication. Subsequently, pulmonary function tests were repeated after 15 minutes of bronchodilator inhalation.

Airway resistance values greater than 1.64 Z scores^14^ and spirometry values lower than -1.64 Z scores^8^ were deemed as altered. The classification of ventilatory disorders followed the recommendations of the ATS/ERS.^13^

Obstructive disorders were considered when FEV_1_/FVC was reduced; and restrictive disorder, when FVC was reduced and FEV_1_/FVC was normal. Mixed disorder was established when both changes simultaneously occurred. The severity of obstructive disorders was defined by the FEV_1_ Z score:

- Mild: Z score≥-2;
- Moderate: -2.5≤Z score<-2;
- Moderate/severe: -3≤Z score<-2.5;
- Severe: -4≤Z score<-3.^16^

A positive bronchodilator response was considered when, in the repetition of the pulmonary function test after bronchodilator application, there was an increase in FEV_1_ and FVC ≥12%^17^ or a reduction of ≥1.25 Z score in the value of airway resistance^18^ in relation to the basal value.

This study was approved by the Research Ethics Committee of Unifesp (#2.131.265). Parents were requested to sign an Informed Consent Form, and children were requested to sign the Assent Form.

The sample size calculation was based on the study conducted by Gonçalves et al.,^3^ considering the 32% prevalence of altered pulmonary function in schoolchildren born preterm, and on the study by Kilbride et al.,^1^ that showed a prevalence of 12% in full-term children. Considering an alpha error of 5%, beta error of 80%, and loss of 20% due to possible problems in the acceptability and reproducibility of pulmonary function curves, the need to include 113 children in each group was noted.

Numerical variables were expressed as mean and standard deviation and were compared by Student’s *t*-test (normal distribution) or Mann-Whitney’s test (non-normal distribution). The assessment of the normality of distribution was verified by the Kolmogorov-Smirnov test. Categorical variables were expressed as number and percentage, and were compared by Chi-square or Fisher’s exact test. Statistical analyses were performed with the *Statistical Package for the Social Sciences* (SPSS) program, version 17 (IBM SPSS Statistics, Somers, NY, United States of America), considering p<0.05 significant.

## RESULTS

A total of 114 children aged 6–9 years in the preterm group and 115 in the full-term group met the inclusion criteria. Of these, two children born preterm and three born full-term were unable to perform spirometry. Thus, in each group, 112 children were studied, and 52 (46.4%) of them were boys. The mean birth weight was 1349±334g and 3182±453g, respectively, in the preterm and full-term groups (p<0.001).

Mothers of preterm children were 29.6±7.3 years old at the time of delivery. During pregnancy, 39 (34.8%) had gestational hypertension; 12 (10.7%), diabetes mellitus; eight (7.1%), hypertension; 30 (26.8%), urinary tract infection; one (0.9%), chorioamnionitis; three (2.7%), placenta previa; and two (1.8%), placental abruption. Antenatal corticosteroid was administered in 62 (55.4%) pregnant women.

The mean gestational age of the preterm group was 30.8±2.8 weeks, and 40 (35.7%) newborns were small for gestational age. During hospitalization in the neonatal unit, 52 (46.4%) preterm infants presented respiratory distress syndrome; 17 (15.2%), patent ductus arteriosus; 23 (20.7%), neonatal sepsis; six (5.4%), meningitis; 37 (33%), periventricular and intraventricular hemorrhage; 19 (17%), retinopathy of prematurity; and 22 (19.6%), oxygen dependency at 36 weeks of corrected age.

In the neonatal unit, 73 (65.2%) preterm infants required invasive mechanical ventilation with a mean ventilation time of 3.2±8.4 days (median=0; q1=0, q3=3). Hospitalization time was 45.3±26.8 days (median=37; q1=26, q3=62).

During childhood until inclusion in the study, the prevalence of asthma diagnosis according to ISAAC,^11^ the presence of wheezing after physical exercise, and the need for hospitalization were higher among premature infants than among those born full-term. In all, 44 preterm infants were hospitalized at least once during childhood. Most hospitalizations were due to respiratory disorders, with 16 hospitalizations due to pneumonia; 16 due to bronchiolitis; three due to asthma; two due to influenza; and the others due to meningitis, seizures, or hernia or phimosis surgeries (Table 1).

**Table 1.** Clinical complications in childhood before inclusion in the study presented by children born preterm and full-term.

	Preterm (n=112)	Full-term^$^ (n=112)	p-value
Bronchiolitis n (%)	29 (25.9)	21/92 (22.8)	0.612*
Pneumonia n (%)	21 (18.8)	8 (7.1)	0.041*
Prior hospitalization n (%)	44 (39.3)	27 (24.1)	0.015*
Number of hospitalizations	0.65±1.3	0.4±0.8	0.075^#^
Asthma diagnosis (ISAAC) n (%)	16 (14.3)	7 (6.3)	0.048*
Wheezing after physical exercise (ISAAC) n (%)	5 (4.5)	2 (2.0)	0.049^&^
Use of bronchodilators n (%)	26 (23.2)	14 (12.5)	0.064*
Use of corticosteroids n (%)	16 (14.4)	7 (6.6)	0.076*
Family history of asthma n (%)	33 (29.5)	18/99 (18.2)	0.056*

ISAAC: *International Study of Asthma and Allergies in Childhood* ^11^; ^$^some parents/guardians were unable to inform about the history of bronchiolitis and asthma in the family; *Chi-square; ^&^Fisher’s exact test; ^#^Mann-Whitney’s test.

At study entry, both groups presented similar anthropometric characteristics (Table 2). In addition, both were similar regarding the adequacy of height-for-age (p=0.155) and body mass index (BMI)-for-age (p=0.177). The height-for-age ratio was adequate in 110 (98.2%) and 112 (100%) children, and the BMI-for-age was adequate for 70 (62.5%) and 74 (66.1%) children. Overweight/obesity occurred in 32 (28.6%) and 33 (29.9%) schoolchildren in the preterm and full-term groups, respectively.

**Table 2.** Children’s anthropometric characteristics at study entry.

	Preterm (n=112) Mean±SD	Full-term (n=112) Mean±SD	p-value
Age (years)	7.7±0.9	7.8±0.9	0.368*
Weight (kg)	27.8±7.9	28.8±7.9	0.265*
Height (cm)	130.0±9.1	130.6±7.6	0.634*
Height-for-age (Z score)	0.03±1.42	0.27±1.41	0.962^#^
BMI (kg/m^2^)	19.5±3.6	20.0±4.3	0.302*
BMI-for-age (Z score)	-0.03±1.87	-0.21±1.67	0.310^#^

SD: standard deviation; BMI: body mass index; *t-test; ^#^Mann-Whitney’s test.

**Figure 1.**
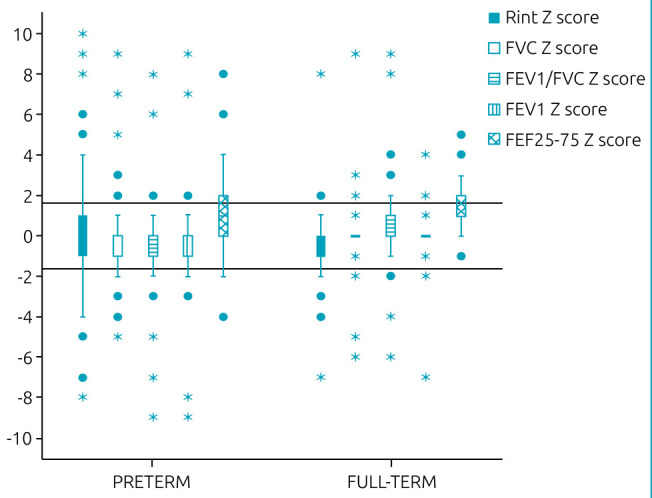
Boxplot with airway resistance values, FVC, FEV_1_/FVC, FEV_1_, and FEF_25-75_ in Z-score values. The two horizontal lines in the center of the figure represent values corresponding to +1.64 and -1.64 Z scores. Note that for airway resistance values, the preterm group has a higher number of children with values above 1.64 Z scores, compared with the full-term group. As for the spirometry parameters, the preterm group, compared with the full-term group, presents greater dispersion of values concerning the median, with a significant reduction in the values of FEV_1_/FVC and FEF_25-75_, indicating obstructive disorder.

The preterm group, when compared with the full-term, presented higher expiratory resistance and lower values of FEV_1_/FVC and FEF_25-75_, in addition to lower % of expected FEV_1_ (Table 3). Figure 1 shows the boxplots of the two groups with values expressed in Z scores for the measured parameters.

**Table 3.** Airway resistance and spirometry values in both groups of children.

	Preterm (n=112) Mean±SD	Full-term (n=112) Mean±SD	p-value*
Rint (KPa/L)	0.67±0.43	0.47±0.15	0.001
% Expected	110.3±73.3	59.5±5.4	<0.001
Z score	0.13±2.24	-1.02±1.29	<0.001
FVC (L)	1.63±0.37	1.69±0.31	0.573
% Expected	95.4±14.8	98.4±11.9	0.279
Z score	-0.39±1.27	-0.15±1.03	0.106
FEV_1_/FVC	87.85±6.91	90.00±6.40	0.013
% Expected	97.7±7.7	100.1±6.9	0.017
Z score	-0.23±1.22	0.14±1.11	0.003
FEV_1_ (L)	1.43±0.32	1.52±0.27	0.655
% Expected	93.6±16.0	98.8±13.1	0.008
Z score	-0.48±1.29	-0.04±1.08	0.071
FEF_25-75_ (L/min)	1.62±0.53	1.90±0.50	0.001
% Expected	86.0±25.6	97.4±22.2	<0.001
Z score	1.16±1.37	2.08±1.26	0.005

SD: standard deviation; *Mann-Whitney’s test; Rint: airway resistance; FVC: forced vital capacity; FEV_1_: forced expiratory volume in one second; FEF_25-75_: forced expiratory flow between 25% and 75% of vital capacity

Rint: airway resistance; FVC: forced vital capacity; FEV_1_: forced expiratory volume in one second; FEF_25-75_: forced expiratory flow between 25 and 75% of vital capacity.

The percentage of children with changes in airway resistance values (16.1 vs. 1.8%, p<0.001) and spirometry (26.8 vs. 13.4%, p=0.012) was higher in the preterm group compared with the full-term group.

Among the children born preterm, nine (30%) presented mild airway restriction; 11 (36.7%), mild airway obstruction; five (16.7%), moderate obstruction; one (3.3%), severe obstruction; and four (13.3%), mixed symptoms of airway restriction and moderate/severe obstruction. Among those born full-term, six (40%) presented mild airway restriction; eight (53.3%), mild airway obstruction; and only one (6.7%), moderate mixed condition (p<0.012).

Regarding airway resistance measures, there was a positive bronchodilator response in 27 (24.1%) and 18 (16.1%) children in the preterm and full-term groups, respectively (p=0.133). For measures of FEV_1_, the positive response was found in 12 (10.7%) children born preterm and in six (5.4%) children born full-term (p=0.198).

Among schoolchildren born preterm, those with altered spirometry, compared with those with normal parameters, presented lower birth weight, higher frequency of oxygen dependency at 36 weeks of corrected age and, during childhood, a higher occurrence of pneumonia and asthma diagnosis and a tendency to higher BMI Z scores (Table 4).

**Table 4.** Demographic and clinical characteristics of the preterm group with and without altered pulmonary function.

	Altered function (n=30)	Normal function (n=82)	p-value
Boys (n=52)	14 (46.7%)	38 (46.3%)	0.976*
Birth weight (g) (n=112)	1.217±310	1398±331	0.011^£^
Gestational age (weeks) (n=112)	30.0±2.8	31.1±2.8	0.065^£^
SGA NB (n=40)	11 (38.7%)	29 (35.4%)	0.244*
Apgar 1^st^ minute (n=112)	6.9±2.3	7.3 ±1.6	0.236^£^
Apgar 5^th^ minute (n=112)	8.3±1.6	8.8±0.7	0.109^£^
RDS (n=52)	18 (60%)	34 (41.5%)	0.082*
Sepsis (n=23)	8 (26.7%)	15 (18.3%)	0.062*
O_2_ dependency at 36 weeks (n=11)	6 (20%)	5 (6.1%)	0.010^&^
Use of mechanical ventilation (n=73)	23 (76.7%)	50 (61%)	0.092*
Days of oxygen therapy in the NICU	15.2±23.6	6.3±17.7	0.002^#^
Current weight-for-age (Z score) (n=112)	-0.07±1.26	0.06±1.76	0.290^#^
Current height-for-age (Z score) (n=112)	0.60±2.21	0.13±0.98	0.414^#^
Current BMI-for-age (Z score) (n=112)	0.63±2.71	0.20±1.40	0.053^#^
History of bronchiolitis (n=29)	11 (36.7%)	18 (22)	0.115*
History of pneumonia (n=21)	10 (33.3%)	11 (13.4%)	0.017*
Asthma diagnosis (ISAAC) (n=16)	8 (26.7%)	8 (9.8%)	0.024*

SGA NB: small for gestational age newborn; RDS: respiratory distress syndrome; O_2_: oxygen; NICU: Neonatal Intensive Care Unit; Current: on the day of the pulmonary function test; BMI: body mass index: ISAAC: *International Study of Asthma and Allergies in Childhood*;^11^ *Chi-square; ^&^Fisher’s exact test; ^#^Mann-Whitney’s test; ^£^ *t*-test.

## DISCUSSION

The present study showed that the preterm group, when compared with the full-term group, presented a higher prevalence of altered pulmonary function with higher airway resistance values and lower parameters of FEV_1_/FVC, FEV_1_, and FEF_25-75_, characterizing pulmonary impairment predominantly of an obstructive nature. Such results have been widely reported in the literature,^1–5^ reinforcing the hypothesis that prematurity has an impact on pulmonary function that may persist into adulthood.^7^

However, it is worth mentioning that the results of studies on pulmonary function testing have varied according to the characteristics of the analyzed neonatal population. In addition to the degree of prematurity, other factors, such as bronchopulmonary dysplasia and mechanical ventilation, have been associated with greater pulmonary impairment,^3,5,7^ similar to what was observed in the present study.

Another variable that may contribute to differences in spirometry results in preterm infants is the choice of the reference standard for interpreting test results.^3,5^ Based on the GLI curves adopted in this study, the prevalence of altered spirometry was 26.8% in schoolchildren born preterm, lower than the 43% found in another study on preterm infants with similar gestational age^3^ when adopting the reference curve of Polgar and Promadhat.^9^ Likewise, Fawke et al.^2^ reported a prevalence of 56% in schoolchildren born extremely preterm considering the reference values recommended by Stanojevic et al.^19^

Conversely, Thunqvist et al.^5^ described, according to GLI standards, a prevalence of alteration in 14% of 6.5-year-old children born with gestational age <27 weeks for FVC and in 23% of them for FEV_1_, evidencing the influence of the reference standard considered, among other possible factors.

In the present study, the higher occurrence of respiratory diseases and wheezing in the past 12 months in the preterm group compared with the full-term group is noteworthy. Furthermore, the authors observed that children born preterm with altered pulmonary function had a higher incidence of childhood pneumonia and wheezing in the past 12 months.

These findings were also described by Thunqvist et al.,^5^ who reported a 40% prevalence of asthma-like disorder in extremely preterm infants compared with 15% of those born full-term at 6.5 years of age. Moreover, preterm infants who reported asthma had lower FEV_1_ Z scores compared with those without such history. These respiratory complications associated with prematurity, whether or not accompanied by a history of bronchopulmonary dysplasia, have been described quite frequently and may explain the predominance of obstructive disorders in individuals born preterm.^3,4,7,20,21^

Yang et al.^7^ also found a predominance of the obstructive pattern in pulmonary function tests in 224 adults aged 26 to 30 years born in the 1986 New Zealand cohort with a gestational age of 28.2±2.5 weeks, compared with 100 full-term controls. In the aforementioned study, a greater severity of the obstructive condition was reported in preterm infants with a history of bronchopulmonary dysplasia and a higher occurrence of asthma among preterm infants compared with those born full-term (34.8 vs. 23%, p=0.034), similar to what was observed in the present study and others.^2,5^

An important clinical implication is that the obstructive pattern may persist into adulthood and clinically manifest as a chronic respiratory disease.^5,7,22^ A systematic review and subsequent meta-analysis of 16 studies showed that, among other factors, prematurity, low birth weight, pulmonary infections, and asthma in childhood are determinants of chronic obstructive pulmonary disease in adults.^23^

In the present study, the prevalence of restrictive changes detected by spirometry was lower than the prevalence of obstructive ones, a result similar to that observed by other researchers,^24^ possibly due to lung growth with increasing age.^25^ Corroborating this result, it is noteworthy that the anthropometric parameters for inclusion in the study were, on average, adequate for age and similar in both groups, justifying the small difference between the prevalence of restrictive disorders among them. Nevertheless, it is worth emphasizing that in the preterm group the percentage of overweight/obese children was similar to the full-term group and also to that of the Brazilian population for the age group from 5 to 9 years old.^26^

This growth profile was also observed in another study.^27^ One possible explanation is that the multidisciplinary follow-up performed at the Premature Outpatient Clinic, with clinical and social support until inclusion in the study, would have contributed to reduce the difference in growth between the groups; however, a need for surveillance of excessive weight gain is observed, as there was a tendency for higher BMI-for-age Z scores among preterm infants with altered pulmonary function, confirming data from the literature that indicate changes in pulmonary function in obese individuals.^28^

This study presents a differentiated peculiarity concerning the reference standards used for interpreting pulmonary function tests. The adopted GLI curves^9^ are considered the international gold standard, because they offer a unified approach for the interpretation of pulmonary function tests, in addition to being multiethnic and validated in many countries,^29^ including Brazil.^30^ Similarly, the inclusion of a control group of full-term schoolchildren and the sample calculation valorize the obtained results.

Conversely, the cross-sectional design of the study, the convenience sample, and the difficulty accessing some data from the full-term group constitute limitations of the study.

Finally, it is worth noting that, although the new reference standard showed a lower prevalence of pulmonary function alteration than that found in previous studies,^2,3^ it is still a high prevalence for a sample of preterm infants with a mean gestational age of 31 weeks. In addition, the finding of higher airway resistance, lower values of FEV_1_/FVC, FEF_25-75_, as well as % of expected FEV_1_, higher prevalence of asthma, and significant proportion of children with positive bronchodilator response among preterm infants suggest the possibility that some of the studied children present a chronic respiratory disease resulting from prematurity.

All in all, this study showed that children aged 6–9 years old who were born preterm assessed by GLI curves showed a higher prevalence of altered pulmonary function, especially those of an obstructive nature, with higher airway resistance and lower spirometry parameters, compared with those born full-term.
